# Impact of Sports on Female Growth and Pubertal Development: A Cohort Study

**DOI:** 10.7759/cureus.75805

**Published:** 2024-12-16

**Authors:** Mariana M Anjos, Carlos Braga, António M Cruz-Ferreira

**Affiliations:** 1 Pediatric Department, Unidade Local de Saúde Médio Tejo, Torres Novas, PRT; 2 General and Family Medicine, Unidade de Saúde Familiar Norton de Matos, Unidade Local de Saúde de Coimbra, Coimbra, PRT; 3 Department of Sports Medicine, Portuguese Rugby Federation, Lisbon, PRT; 4 Centre for Health Studies and Research of the University of Coimbra, University of Coimbra, Coimbra, PRT; 5 Faculty of Medicine, University of Coimbra, Coimbra, PRT; 6 Department of Sports Medicine, Portuguese Institute of Sport and Youth, Coimbra, PRT

**Keywords:** athletes, exercise, female athlete triad syndrome, growth, puberty, sexual maturation

## Abstract

Introduction

The participation of women in sports is increasing, and the rising training demands may impact growth and pubertal development. High-intensity sports are often linked to delayed growth and bone maturation due to energy deficits and intense regimens. These factors may increase the risk of injury and musculoskeletal issues. Additionally, elite athletes face higher incidences of eating disorders and the female athlete triad, affecting both physical and mental health.

This article aims to examine the impact of various sports on female physiological development, particularly during puberty.

Methods

This retrospective observational study was conducted over six months and included female athletes aged 16-30 years involved in sports. Participants provided online consent. Data were collected through a self-administered electronic questionnaire, covering sociodemographic factors, menstrual cycle characteristics, growth patterns, pubertal development, lifestyle habits, and perceived effects on athletic performance. Responses, gathered using Google Forms® (Google LLC, Mountain View, California, United States), were analyzed using the IBM SPSS Statistics for Windows, Version 20 (Released 2011; IBM Corp., Armonk, New York, United States).

Results

The study included 121 female athletes (mean age: 20 years), with rugby (n=35), gymnastics (n=23), and soccer (n=22) as the most common sports. Participants generally began sports around age 11, with gymnastics athletes starting earlier than those in other sports. The median weekly training duration was six hours across three sessions, with most athletes competing nationally (n=82) or internationally (n=29). The median age at menarche was 12 years, occurring later in soccer, gymnastics, and triathlon athletes. Rugby and soccer players demonstrated greater height increases post-menarche, although seven athletes, primarily rugby players, did not reach their target familial height. Achievement of target height was significantly associated with the number of weekly training hours (p=0.019).

Dietary restrictions were most common among rugby (n=7), gymnastics (n=6), and soccer (n=5) athletes. Eating disorders, primarily anorexia and bulimia, were observed in 11 athletes, particularly in rugby and gymnastics, and correlated with the age of thelarche. Injuries, notably in rugby and gymnastics, were frequent, and injury incidence was associated with the onset age of eating disorders.

Conclusion

This study highlights how intensive training, low-energy diets, and eating disorders negatively influence growth, pubertal development, and injury rates in female athletes. Heavy training loads combined with low-energy diets or eating disorders delay puberty, impair growth, and heighten injury susceptibility due to hormonal imbalances.

## Introduction

The participation of women in sports has been steadily increasing, paralleling the rising demands of competitions, increased training loads, and the continuous pressure on athletes [1]. The growth process is complex and dynamic, influenced by a variety of genetic and environmental factors [2]. Participation in sports during childhood and adolescence can impact growth and pubertal development, depending on the type of exercise, intensity, frequency, duration, and the age at which the sport is initiated [3]. Major sports requiring intensive physical training during childhood and adolescence, such as gymnastics (both rhythmic and artistic), swimming, rowing, wrestling, track and field, and tennis, often involve high training intensity during growth periods; these demands are associated with reduced energy intake and increased energy expenditure, potentially impacting bone maturation and leading to reduced genetic growth potential or delayed catch-up growth [4,5]. This may also increase the likelihood of injuries, resulting in higher rates of musculoskeletal injuries among athletes [2].

Relative energy deficiency in sport (RED-S) is characterized by low energy availability, which may result from low-energy diets, whether intentional or unintentional, or from excessive exercise [4,6]. RED-S leads to alterations in several hormonal pathways, with energy redistribution favoring the preservation of vital systems [4]. One of these changes is a decrease in the insulin-like growth factor-1 (IGF-1) axis, which mediates many growth hormone (GH) functions and is responsible for the GH resistance observed in RED-S [4,6]. Furthermore, RED-S poses a risk of bone mass loss, potentially preventing individuals from reaching peak bone mass [4].

Puberty is a multifaceted phase characterized by rapid growth and the development of secondary sexual characteristics [7]. These physical changes are largely influenced by hormonal levels [1]. Additionally, there is a high incidence of eating disorders among elite athletes, with growing concern around the recognition of the female athlete triad, characterized by eating disorders, amenorrhea, and osteoporosis [4]. Dissatisfaction with body image, physical appearance, or sports performance can lead to the development of eating disorders in athletes, increasing their vulnerability to a variety of mental health issues [8,9].

Currently, there is growing concern about women's sports, leading to an increase in research focused on women's sports health and performance [2,7]. This article explores the multifactorial impact of sports on female growth and pubertal development.

Objectives

To determine the effects of sport type, training load, and age at training onset on growth and pubertal development. To assess the prevalence of dietary restrictions and eating disorders and their impact on pubertal development and injury incidence.

## Materials and methods

Study design

Retrospective observational study conducted over a six-month period. The study was conducted from June 2023 to December 2023. The study took place at Unidade Saúde Familiar Norton de Matos, Unidade Local de Saúde de Coimbra, Coimbra, Portugal.

Participants

Inclusion Criteria

Female athletes aged between 16 and 30 years participating in dance, gymnastics, athletics, swimming, rugby, soccer, and other sports who voluntarily consent to participate in the study by completing a written informed consent form (via online registration).

Exclusion Criteria

Participants with incomplete questionnaire responses.

Ethical approval

The study was conducted according with the principles of the Declaration of Helsinki, submitted to the Ethics Committee of our institution (approval no. 72/2023) and obtained a favorable opinion from the respective committee. Written informed consent was obtained from the participants before study initiation. 

Data collection

Data was collected through a self-administered electronic questionnaire, which included sociodemographic details, menstrual cycle characteristics, growth and pubertal development, lifestyle habits, and their impact on athletic performance (Appendix 1). Data collection was facilitated through Google Forms® (Google LLC, Mountain View, California, United States) platform, targeting sports organizations and clubs.

Data analysis

The questionnaire responses were compiled into an Excel® file for analysis. Statistical analysis and comparison of the results were performed using IBM SPSS Statistics for Windows, Version 25 (Released 2017; IBM Corp., Armonk, New York, United States).

To evaluate differences in key variables across sports categories, chi-square and a one-way analysis of variance (ANOVA) were used. Post-hoc comparisons were conducted using Tukey's honestly significant difference (HSD) test to identify specific group differences where ANOVA results were significant. All analyses were conducted at a significance level of α=0.05.

## Results

A total of 121 female participants were included, with a mean age of 20 years (range: 16-30 years, median=20). The most commonly practiced sports were rugby (n=35), gymnastics (n=23), and soccer (n=22) (Table 1). Sports with the fewest participants were athletics (n=12), swimming (n=6), triathlon (n=6) and roller hockey (n=4). The “Other” sports category included crossfit, sailing, and handball, as well as one case where the type of sport practiced was not specified. 

**Table 1:** Characterization of the different sports

	Swimming	Athletics	Rugby	Roller Hockey	Soccer	Gymnastics	Triatlhon
Number of athletes	6	12	35	4	22	23	6
Median age at sports onset	8.5	12	15	6.5	11.5	6	8.5
Median workouts per week	9.5	10	4.5	5	4.5	9	14.5
Median age at menarche (range)	12 (12-14)	12 (11-15)	12 (9-17)	12 (12-13)	13 (8-16)	13 (11-16)	13 (11-15)
Median age at thelarche (range)	12.5 (11-14)	11.25 (9-15)	12 (7-14)	11 (9-12)	12 (10-16)	12 (8-16)	12 (12-14)
Median age of puparche (range)	12 (9-14)	11.5 (9-15)	11 (8-14)	11 (9-13)	12 (9.5-15]	12 (8-18)	12 (10-14)

The median age at which participants began practicing sports was 11 years (range: 2-24 years), with a median duration of sports practice of 9.5 years. Regarding the onset of sports practice, there was a statistically significant relationship (p<0.001) between the different sports, indicating that gymnastic athletes tend to start earlier than those in athletics, rugby, or soccer. Statistical analysis revealed that gymnastics athletes have significantly more years of practice compared to rugby players. 

Overall, participants engaged in a median of six hours of training per week (range: 1.5-20 hours), distributed over a median of three training sessions per week (range: 2-19). The majority of athletes competed at the national level (n=82), while 24% participated in international competitions (n=29). No statistically significant relationship was found between years of practice or the number of weekly training hours and the level of competition.

The median age at menarche was 12 years (range: eight to 17 years). Menarche occurred later in soccer, gymnastics, and triathlon (median=13 years) (Table 1). No statistically significant relationship was found between the level of competition and the age at menarche, age at thelarche, or age at pubarche. 

On average, athletes experienced an approximate increase in height of 9 cm from menarche to their current age (range: 0-37 cm), with more pronounced growth observed in rugby and soccer (Table 2). In this sample, seven athletes did not reach their target family height, six of whom were rugby players who trained a median of five hours per week (range: 3-7.5 hours). Target height achievement was significantly associated with the higher number of weekly training hours (p=0.019). No other variable showed a statistically significant relationship with the attainment of target height. 

**Table 2:** Increase in height from menarche to their current age in different sports

	Swimming	Athletics	Rugby	Roller Hockey	Soccer	Gymnastics	Triatlhon
Median increase in height (cm) (range)	5 (2-24)	7 (1-17)	10 (2-35)	9.5 (6-30)	12 (1-27)	8 (0-22)	10 (0-18)

The majority of athletes did not have any diagnosed chronic illnesses (n=101). During sports practice, 41 athletes were on chronic medication, with oral contraceptives being the most common (n=27).

Overall, most young athletes did not use supplements during their sports practice (n=80). Among those who used supplements (n=38), the majority took vitamins or minerals (n=20), followed by protein supplements (n=11) and creatine (n=10).

In the analyzed sample, 30 athletes (24.8%) followed some form of diet or dietary restriction during sports practice. Eleven athletes (9.1%) were diagnosed with eating disorders, most commonly anorexia nervosa or bulimia (n=6). The sports most frequently associated with anorexia nervosa or bulimia were rugby (n=4) and gymnastics (n=2), though no statistical association was found (Table 3). The median age at which the eating disorder was identified was 15 years. Eating disorders were statistically associated with the age at thelarche (p<0.001).

**Table 3:** Characterization of eating disorders according to sports

	Anorexia/bulimia	Binge eating	Body image disorder
Rugby (n)	4	1	0
Gymnastics (n)	2	0	2
Athletics (n)	0	1	0
Triathlon (n)	0	1	0

Dietary restrictions were more commonly observed in rugby (n=7), gymnastics (n=6), and soccer (n=5), although these associations were not statistically significant. A statistical relationship was found between years of practice (p=0.045), higher weekly training hours (p=0.038) and age at thelarche (p=0.041) with the presence of dietary restrictions among the athletes. There was no statistical association found between dietary restrictions or eating disorders and the achievement of target height.

A total of 40 athletes were identified with secondary amenorrhea in the sample, 12 of whom were following a diet or dietary restriction during sports practice, and seven had concurrent eating disorders. The median duration of secondary amenorrhea was three months (range: one to 12 months).

The majority of athletes experienced some form of injury during their sports practice (n=87), with a median of two injuries (range: 1-20). Injuries were most commonly reported in rugby (n=25), gymnastics (n=20), soccer (n=16), and athletics (n=12). A significant correlation was found between the number of injuries throughout an athlete’s career and the age of onset of an eating disorder (p=0.006). 

## Discussion

Linear growth reaches its exponential peak during adolescence and may be influenced by both environmental and genetic factors [5]. In this study, the average age at which athletes began practicing sports was 11 years, with an average duration of 9.5 years of practice, representing a significant period of sports involvement during the pubertal phase of young female athletes. Previous studies have shown that training during this critical period can impact physical growth and pubertal maturation [5,10]. Additionally, in this study, gymnastics started at a younger age and had more years of practicing compared to athletes in other sports, suggesting a greater potential for affecting growth in this group. 

Target height achievement is known to be influenced by the intensity, frequency, and duration of exercise [5]. In this study, seven athletes, six of whom were rugby players, did not reach their potential height. A statistically significant relationship was found between target height achievement and the higher number of weekly training hours, supporting previous findings stating that both intensity and frequency of sports practice can impact growth [5]. However, in this study, the duration of sports practice did not correlate with target height achievement as reported in prior research. This may explain why gymnastics, despite being the sport with the most years of practice, did not have a significant representation among athletes who failed to reach their target height. Furthermore, the majority of individuals in this study competed at the national level, likely reflecting a lower training load than required to affect potential growth.

Relative energy deficiency in sport (RED-S) results from low energy availability due to diet or exercise, leading to hormonal changes, growth hormone resistance, and risks like impaired bone mass development [4,6]. There was a 24.8% prevalence of dietary restrictions in this study, which is consistent with the prevalence of low energy availability reported in previous studies (22-58%) [11]. In this study, a statistically significant relationship was found between years of practice (p=0.045), higher weekly training hours (p=0.038), age at thelarche (p=0.041), and the presence of dietary restrictions. Additionally, 9.1% of athletes in this study presented with eating disorders, which aligns with the prevalence rates reported in the literature, varying between 2 and 42%, depending on the sport [12]. Eating disorders were significantly associated with the age at thelarche (p<0.001). Therefore, this study demonstrates a correlation between low-energy diets, heavy training loads, and delayed puberty, with the strongest effects observed in the presence of eating disorders, likely due to the altered hormonal pathways resulting from the combination of these factors. 

RED-S is a known risk factor for stress fractures, as is a heavy training load [3]. The presence of an eating disorder and its age of onset appeared to play a significant role in the incidence of injuries, as a statistically significant relationship was found between the two in this study. 

This study is likely limited by the use of a self-administered questionnaire, which assumes the accuracy of the information provided by the participants. Therefore, more controlled observational studies are recommended to further explore these issues. 

## Conclusions

This study highlights the multifaceted impact of sports practice on young female athletes' growth, pubertal development, and increased risk of sports injuries.

Heavy training loads, particularly when combined with low-energy diets and eating disorders, were found to delay pubertal milestones, affect growth potential, and increase susceptibility to stress injuries. Furthermore, dietary restrictions and eating disorders, prevalent in a significant proportion of athletes, were associated with delayed puberty and increased injury rates, highlighting the influence of altered hormonal pathways due to energy imbalances.

Early identification and monitoring of athletes with these risk factors are crucial to mitigating long-term health consequences. Implementing appropriate management strategies with supportive environments can help protect the long-term health of female athletes and maximize the positive impact of sports on physical and mental health.

## Appendices

Appendix 1

Questionnaire - Effect of the Menstrual Cycle and Contraception on Female Sports Practice in Youth

The participation of females in sports has increased significantly, paralleling the growing demands of competitions, increased training loads, and the constant pressure placed on athletes.

In this context, the research team at the Norton de Matos Family Health Unit is conducting a study titled “Menstruation and Contraception in Portuguese Sports.”

This study was previously approved by the Ethics Committee of the Regional Health Administration of the Center, receiving a favorable opinion from the respective committee.

The primary objective of this study is to determine the effect of sports practice on the health of young female athletes.

Please note that participation is entirely voluntary, and you may withdraw at any time without providing any justification or incurring any negative consequences. Additionally, at no point will we ask for your name; only the project researchers will have access to your responses. The researchers commit to:

a) ensuring complete confidentiality regarding the data collected, as all information will be stored in a database that prevents any direct or indirect identification of participants. This survey is anonymous; no identifying information will be requested, and we guarantee full confidentiality of your information. Data collected is solely intended for academic and research purposes, and responses will be used only for statistical analysis.

b) using the data provided by participants strictly for research purposes. Results will be considered only in aggregate form and processed solely as collective data.

Therefore, participation in this study is entirely voluntary, and declining to participate will in no way affect the quality of medical care you receive. No immediate personal benefit is offered by participating in this study, nor will it incur any financial cost. Should you wish, more detailed scientific information on the topic will be provided, and any questions you may have will be promptly addressed by the research team.

The research team will not receive any financial compensation for the work involved in this study.

This survey is intended for athletes or former athletes between the ages of 16 and 30.

The average time required to complete the questionnaire is approximately eight minutes.

For any questions or concerns, please feel free to reach out.

Mariana M. Anjos/Carlos Braga

1. Do you consent to participate in this survey? Please select one option. ○ Yes ○ No

2. I understand the objectives of this survey, as well as the verbal information provided, and I freely and willingly consent to participate. Please select one option. ○ Yes ○ No

3. I have been informed that I am free to refuse participation in this survey without any effect on the assistance I receive. Please select one option. ○ Yes ○ No

4. Considering that anonymity is guaranteed, I consent to the use of the provided data for statistical analysis and to the sharing of study results with the scientific community. Please select one option. ○ Yes ○ No

General Information

5. Age?

6. Year of Birth?

7. Sport Practiced?

8. Age at Start of Sports Practice?

9. Years of Sports Practice?

10. Average Training Hours per Week?

11. Number of Training Sessions per Week?

12. What type of competitions did you participate in? Select all applicable options. □ Local □ National □ International □ Elite

Menstrual and Pubertal Development

13. Age at First Menstruation?

14. Age at Breast Development?

15. Age at Appearance of Pubic Hair?

16. Bone Age? (If unknown, please skip to the next question)

17. Average Weight During Sports Practice (kg)?

18. Current Weight (kg)? 

19. Current Height?

20. Height at First Menstruation?

21. Mother's Height?

22. Father's Height?

Personal History

23. Usual/Chronic Medications During Sports Practice?

24. Supplements Used During Sports Practice?

25. Known Previous Illnesses?

26. Have you ever followed any diet or dietary restriction? Please select one option. ○ Yes ○ No

26.1. If yes, please specify the diet or restriction.

27. Have you ever experienced an eating disorder? Please select one option. ○ Yes ○ No

27.1. If yes, what type of eating disorder?

27.2. At what age did you experience this eating disorder?

28. Have you experienced any injuries during sports practice? Please select one option. ○ Yes ○ No

28.1. If yes, how many injuries?

28.2. If yes, what type of injuries did you experience?

Gynecological History

29. Have you ever been pregnant? Please select one option. ○ Yes ○ No

30. Age(s) at Start of Pregnancy

31. How many times have you been pregnant? Please select one option. ○ 0 ○ 1 ○ 2 ○ 3 ○ 4 ○ More than 5 times

32. After your first menstruation, did you ever stop menstruating? Please select one option. ○ Yes ○ No

32.1. If yes, how long did you stop menstruating?
